# Exploring Western Australian Women’s experiences of sharing positive birth stories

**DOI:** 10.1186/s12884-022-05226-5

**Published:** 2022-12-28

**Authors:** Zaneta Ann Neucom, Kelly Johanna Prandl

**Affiliations:** 1 Curtin University, Kent Street, Bentley, WA 6102 Australia; 2 Clinical Psychologist MACPA, Curtin University, Kent Street, Bentley, WA 6102 Australia; 3 Currently: Hyde Park Psychology, HIGHGATE, 500 William Street, WA 6003 Australia

**Keywords:** Positive birth, Birth stories, Childbirth, Social identity theory, Need-to-belong theory

## Abstract

**Background:**

Fear can impact childbirth experiences, yet most birth stories women hear portray birth as unfavourable, and women must actively seek out positive birth stories.

**Aims and objectives:**

We aim to explore how women feel when sharing positive birth stories and the socially constructed motivations for or against sharing. Research question: What are the experiences of women who share positive birth stories?

**Methods:**

A qualitative descriptive approach was adopted, adhering to Standards of Reporting Qualitative Research (SRQR) guidelines, and underpinned by an interpretivist research philosophy. Participants were recruited via Facebook using a non-probability, voluntary-response, purposive sampling method. Twelve English-speaking Western Australian women aged 24–38 years identified as having had a positive vaginal birth experience within the past 5 years. Semi-structured interviews were transcribed verbatim and analysed using thematic analysis.

**Findings:**

The theme Not Safe to Share and sub-themes The Perils of Sharing and Changing the Narrative explores how participants repeatedly felt unable to share their birth stories because society’s dominant view of childbirth was negative. It describes the experienced or anticipated reactions that contributed to feeling unsafe and how participants withheld or altered their stories to feel accepted. The theme Safe Spaces and sub-theme The Joys of Sharing, explored participants’ love of sharing their birth stories in safe spaces, allowing re-access to feelings of elation, validation of their stories, and opportunity to empower others.

**Conclusions:**

Women often feel reluctant to share their positive birth stories. Findings may help understand the lack of availability of positive birth stories in our society. Normalising the positive birth experience may improve the experience of sharing positive birth stories, potentially improving society’s view of childbirth.

## Background

Birthing a baby is one of the most varied, unique, and life-changing experiences a woman may encounter in her lifetime. A woman’s experience of labour and delivering a baby can be influenced by her perceptions of childbirth, informed by things such as antenatal education [1], media exposure [2], and other women’s birth stories [3]. A negative portrayal of childbirth, commonly shared among women and depicted in public media, can lead to fear of childbirth [2]. Fear of childbirth can negatively impact birth experiences [4], creating a self-fulfilling prophecy that perpetuates the negative portrayal of childbirth. Despite this, hearing women share positive birth stories seem relatively rare, and pregnant women who want to hear other women’s positive experiences appear to need to actively seek them out [3].

## Fear in childbirth

Available research has found that many women fear childbirth, afraid of complications for self or baby, inadequate or absent care by midwives or doctors [5], experiencing labour pain, interventions in birth, or fear of the unknown [6]. Fear of childbirth can range from low to severe, and severe cases are referred to as tokophobia, which is the “unreasoning dread of childbirth” [7](p4). This fear may result from previous labour length and interventions during previous deliveries [4], previous birth trauma [8], or from hearing other women’s “horror stories” [5](p69). Research has observed many first-time mothers often build an image of childbirth based on other women’s birth stories, antenatal information, internet sources, childbirth education books, and what they see on television [1, 2, 9–12].

Fear can be incited by the negative representation of childbirth, portrayed on television shows as excruciating, terrifying, and often depicts a woman, covered in sweat, screaming, laying on her back in a hospital bed, being tended to by an obstetrician [12]. The top 10 best-selling pregnancy books in the United States all state pain as a given, describing it from something to endure, to excruciating and needless suffering [9]. A review by Luce et al [2], investigated the portrayal of childbirth in the media and found it perpetuated the view of childbirth as a medical event. Depictions emphasised the specialist doctor’s involvement with little-to-no midwife contribution, moving away from childbirth as a natural event towards pathologising it, and hence removing power from the birthing woman [2].

The medicalisation of childbirth began in the seventeenth century with the introduction of educated doctors outranking midwives, whose knowledge from experience had been passed on from other midwives [13]. Women were encouraged to birth in hospitals where scientific knowledge and ongoing education of obstetricians was prioritised as superior to midwives experiential knowledge of birthing [14]. Feminist movements at the time fought for women’s rights to birth in the perceived safety of hospitals with access to pain relief [15]. Birthing in hospitals left women susceptible to contracting puerperal fever and infection, due to the lack of knowledge at the time of the importance of sterilisation and handwashing, which led to a higher rate of perinatal mortality [14]. No longer considered a natural process, childbirth was framed as dangerous and in need of intervention [16]. This meant controlling birthing positions for the convenience of medical staff, introducing pain relief, using sometimes-unnecessary interventions, and the introduction of complying to institutional (hospital) rules, which all lead to a loss of agency and control for the birthing mother [16]. Interestingly, in an analysis of the top 10 best-selling childbirth education books in the United States, only three books portrayed the woman as an agent capable of making sound decisions regarding her own birth experience [9]. Women who feel in control of their birth and their bodies during childbirth are more likely to have a more positive birth experience and extinguish previously held fear [17].

Fear of childbirth can impact birth outcomes by increasing the rate of elective and emergency caesarean-section [18], or increasing the length of labour [4]. Fear of childbirth can also impact a woman’s psychological well-being and is associated with increased levels of post-traumatic stress disorder, anxiety, and depression [19]. Women who fear childbirth report poorer attachment with their baby, which can impact adjustment to motherhood, and breastfeeding outcomes [20, 21]. Fear may also impact women’s future reproduction [22] and birth experiences [8, 23].

## Birthing without fear

An experimental study by Ucar and Golbasi [24], assessed whether expectant mothers could combat their fear of childbirth by participating in antenatal childbirth education programs based on a cognitive behavioural therapeutic approach. The program included questioning automatic negative thoughts, information and demonstrations of childbirth, using the ABC model (Antecedents-Behaviour-Consequences: identifying antecedents, beliefs, and consequences of an event), and relaxation methods and practice [24]. They found that women who participated in this program reported significantly lower levels of fear prior to childbirth, shorter labour duration, lower pain scores during labour, and self-reported more positive birth experiences when compared to women in the control group who did not participate in this program [24]. Antenatal education may reduce the fear of childbirth for pregnant women [25, 26], particularly for women who had previously recorded high levels of fear [27]. However, Sanders and Crozier [10] argue that antenatal classes are not helpful for all women, stating that for some, the volume of information provided in antenatal classes can be overwhelming, and knowledge of possible complications may induce fear. It may also depend on the type of antenatal education, according to Streibert et al [28], who found midwife-led antenatal education was unsuccessful in enabling women to release fear, compared to the success of a self-hypnosis focused antenatal education. A review of the impact of antenatal education on birth outcomes found that participation in antenatal education improved maternal stress and self-efficacy and lowered the rate of caesarean section and epidural use [29]. However, this review did not include a measure of fear and therefore cannot determine whether a reduction of fear contributed to these improved outcomes [29]. The heterogenous nature of antenatal education makes it difficult to ascertain the impact on the myriad of birth outcomes, including fear in childbirth, and available literature appears inconsistent [29–31]. It may depend on whether the education programs and people who deliver them portray a positive view of childbirth on how successfully it reduces fear.

## Positive births

The term “positive birth” is subjective and may mean different things to different women, from the birth of a healthy baby through to feelings of euphoria, it is an appraisal made by the mother regardless of mode of birth and can include (but is not limited to) homebirths, caesarean sections, and medically managed births. Caregiver support is a significant contributor to a positive evaluation of childbirth, and women need to feel connected to their care provider through being heard, supported, and through continuity of care [32]. To achieve a positive birth, women need to feel safe, secure, and supported [33], in a culturally appropriate manner [10]. Women who felt supported during their labour and antenatal period recorded less pain than women who did not feel supported, resulting in a self-reported positive birth [34]. Women who have had previous birth trauma are particularly vulnerable, but having their choices supported by midwives or caregivers enables them to feel empowered and in control of their births [8].

A Swedish study on positive births by Karlstrom et al [35], also found that external factors such as safety, support (from both midwife and support person), and control, led to very positive birth experiences. They also attributed positive births to internal factors such as women feeling empowered, strong and in control, and trusting themselves and their bodies [35]. Participants in this study spoke about positively anticipating childbirth due to inspiration from crucial female role models, such as mothers or grandmothers, illustrating the importance of hearing positive birth stories. According to one participant, entering childbirth with a positive mindset helped facilitate a positive outcome, and mental preparation, practise, and training was imperative [35]. All participants recorded a positive birth experience, importantly not without pain; all felt they were in charge, taking an active role in their birth, and were involved in decision making [35]. Women who perceive their births as positive report “falling in love” with their baby sooner and more positive self-reported parenting scores 8 months after birth ([36] p616).

## Sharing birth stories

Birth stories “possess a capacity to shape and reshape expectations of childbirth” ([37] p8). However, Kay et al [12] found that although it shapes expectations, sharing birth stories does not contribute to meaningful knowledge on childbirth. Dahlen et al [36] found that sharing birth stories served as an initiation into what they called “motherland”, a place where women can support each other, sharing what they refer to as “motherwisdom”*,* to understand the shared challenges of motherhood [3]. Grimes et al [38] found that birth stories are an accessible and popular source of information for pregnant women. Women are more likely to rely on birth stories from family and friends than the internet or antenatal education classes [38]. Johnson et al [3] explored factors that motivate women to share their birth stories and found that many women shared and listened to stories as a means of determining what is considered “normal”. They also found that many women share birth stories as a form of processing, particularly for traumatic births, to validate their experience. Indeed, sharing stories to validate and process traumatic births appears common practice [3, 36, 39], and can be very beneficial for the woman sharing her story [40]. What is the impact of these stories for the listener on perpetuating fear of childbirth? One woman compared sharing birth stories to Amazon reviews stating, “some people will put up good reviews but most of the people who are making the effort to put a review on is because it’s negative” ([12] p4). Hearing inspirational and positive stories can empower women to believe they are capable, and enter birthing with a positive outlook [10], yet consistently research has observed women are more likely to share negative birth stories [12]. Kay et al [12] found that women who birthed 40–50 years ago reported not feeling comfortable sharing positive birth stories as it may make other women feel like they had failed. More recently, some women who heard positive birth stories dismissed them as being inaccurately retold, failing to believe childbirth could be so different from the idea of the sweaty, painful media portrayal or horror stories heard from other women [12]. Is the initiation into motherland contingent on the normative negative story as a rite-of-passage?

## Birth stories from a theoretical perspective

Perhaps the choice not to share positive birth stories can be explained by Need-to-Belong Theory, which posits that people adapt their behaviours and stories to seek approval from, and therefore connection to, others [41]. Could it be that women who have positive birth stories adapt their stories or selectively recount them to feel they belong to a society dominated by a negative view of childbirth? Similarly, Social Identity Theory suggests that individuals define themselves as belonging to a group rather than as unique or different [42]. People evaluate themselves and others with similar characteristics as ingroup members and those with different characteristics as outgroup members [42]. If applied to sharing birth stories, one could argue that traumatic or painful births that align with birthing norms available in the media and other’s birth stories may offer ingroup membership to motherland, and similarly positive birth stories create an outgroup status. Perhaps those reactions from others may account for why women need to actively seek positive birth stories [3].

## The current study

The myriad of research describing the impact of fear in childbirth supports the idea of encouraging a more positive view of childbirth [4, 19, 21]. The motivation for women to share their traumatic birth stories appears to be understood; however, no research exploring women’s experiences surrounding sharing of positive birth stories was identified. Understanding how women feel when sharing positive birth stories and how others react to hearing them may illuminate the socially constructed motivations for or against sharing these stories. Using a social constructivist framework, we aim to qualitatively explore: what are the experiences of women who share positive birth stories? As the term positive birth is subjective, it will be self-determined by how the women perceive their births for this study. Results may elucidate areas for focus in future research on sharing birth stories and releasing the fear of childbirth, which serve to encourage women to release fear of childbirth and feel empowered to strive for a positive birth experience, and in doing so, improve childbirth experiences and outcomes in the future.

## Methods

### Research design

Ethics approval was obtained from Curtin University Human Research Ethics Committee (HRE2021–0270), and we offered no incentives for participation. A qualitative, descriptive design was used to align with our social constructivist epistemology. Social constructivism is the belief that an individual’s knowledge is constructed through their interactions with others and the environment within the context of their previously held knowledge and experiences [43].

This research was inspired by the lived experience of the first author (ZN) in sharing her positive birth story. She had a positive birth following two traumatic birth experiences. She felt other women did not want to hear about her positive birth, based on an adverse reaction of disbelief or aversion, which became a barrier to sharing her positive story. To manage preconceived understandings of sharing positive birth stories, the researchers kept a reflexive journal and audit trail, kept interview questions open and neutral, and actively sought alternative points of view through discussions with women and midwives.

### Recruitment and eligibility

We recruited participants by posting an advertisement on Facebook in May 2021, that was shared by friends, including one hypnobirthing instructor, a midwife, and a maternity clinic. Participants emailed to express their interest in participating and were contacted via return email and telephone to determine eligibility, forward the Participant Information Form and Consent Form, and set an interview time. ZN removed the advertisement after 5 days, having received 61 emails from women offering participation. We did not interview women with whom we have existing relationships.

Employing a non-probability, voluntary-response, purposive sampling method, ZN recruited and interviewed 12 Caucasian women aged between 24 and 38 years (*M* = 34.00, *SD* = 3.79), who had each given birth between one and four times (*M* = 2.25 births each, see Table 1)). All participants resided in Western Australia, were currently married or in a committed relationship, and had tertiary educations ranging from Technical and Further Education (TAFE) qualifications to post-graduate university degrees. Women were required to be living in Australia, over 18 years of age, and speak English as their first language to be eligible for this study. Eligibility also included women identifying as having had a positive vaginal birth experience within the past 5 years, which aligned with the timeframe used in Johnson et al. [3] Birthing experiences were varied and included homebirths, births with no intervention, midwifery-led care, obstetric-led care, public and private hospital births, epidural or gas pain relief, inductions, episiotomies, and vacuum extraction. No participants opted out of the study at any time.

**Table 1:** Participant Demographic Information

Participant demographics	*N*(%)	*M*(*SD*)
Cultural background:		
Caucasian	12(100)	
Age		34(3.79)
Number of births: *		
One birth	3(25)	
Two births	5(41)	
Three births	2(17)	
Four births	2(17)	
Education:		
TAFE	1(8)	
Undergraduate degree	3(25)	
Post-graduate degree	8(67)	
Relationship status:		
Married	10(83)	
In a committed relationship	2(17)	
Single	0(0)	
Most recent birth details:		
Home birth	3(25)	
Hospital birth	9(75)	

Note:* Includes stillbirth

We considered Malterud et al [44] five dimensions for research sampling. Given the narrow aim of the study, the potential for similarities in our sample, researcher experience, and the theoretical underpinning, we found that 12 participants provided adequate data for quality, information power, and richness of data.

### Data collection

Interviews were conducted face-to-face, three at a mutually convenient local café, and nine at private residences. Interviews often included the participant’s children, and one interview included the woman’s husband. ZN conducted all interviews, ranging from 11 to 74 minutes, with an average interview time of 44.33 minutes. Interviews were digitally audio-recorded on two devices.

Participants completed a paper-based demographic survey that included age, number of births, year of positive birth, marital status, ethnicity, and educational background, like that used in Johnson et al. [3] We used a semi-structured interview with open-ended questions designed based on guidelines offered by Callister [39], and questions used by Rodriguez-Almagro et al. [45] Questions are presented in Table 2 and included: “How do you feel when sharing your birth story?” and “tell me about situations where you felt comfortable sharing your birth story.” Additionally, prompts such as “how did you feel when that happened?” or “what did you think about that?” allowed richer data. As data emerged from initial interviews, we added the question “what motivated you to participate in this research?” as answers may illuminate nuances in the phenomenon. ZN informed participants that she would not talk much throughout the interviews, as this appeared to unnerve initial participants. ZN debriefed them with the motivations for the research at the end of the interview to avoid skewing the data. ZN transcribed interviews verbatim into Microsoft Word, applying pseudonyms to people and redacting any identifying information to de-identify data. ZN emailed each participant an interpretive summary of their interview, a member-checking process to improve the credibility of the research [46, 47], and all participants confirmed an accurate interpretation. Data were then transferred into Microsoft Excel for analysis.

**Table 2:** Semi-structured Interview Questions

**Semi-structured interview questions:**	
What motivated you to take part in this research?	
Tell me about your positive birth experience.	
Why do you consider your birth story positive? What aspects of your birth make it a positive birth?	
How did you feel after your birth?	
How have these feelings changed over time?	
Tell me about situations where you have felt comfortable sharing your birth story.	
Tell me about a situation where you have felt uncomfortable sharing your birth story?	
Tell me about other people’s reactions on hearing your birth story.	
How do you feel when sharing your birth story?	
Tell me about a time you may have chosen not to share your birth story.	
How do you feel when you hear someone else’s birth story?	
**Prompts for further information:**	
How did that feel?	
What did you think when that happened?	
Can you tell me more about that?	

### Data analysis

A thematic analysis based on guidelines by Clarke et al [48], provided a rich description of women’s collective lived experiences sharing their positive birth stories whilst allowing a theory-based interpretation to help understand that experience within a social constructivist framework. ZN familiarised herself with the data whilst conducting interviews, transcribing, and re-reading transcripts, and adopted a semantic, inductive approach to identify codes and patterns in the data [48]. Coding was also performed on part of two transcripts by the research team to enhance trustworthiness and found similar codes were generated. Patterns in the data were initially identified during the interviews and developed during transcribing and analysis, and codes were then organised into developing themes. ZN generated names and descriptions for each theme based on language used in the data, supporting each theme with key quotes in preparation for writing the findings. ZN created a thematic map and checked themes against the data set, before attempting to apply the meanings of those themes within the context of the research question [49]. Themes were also discussed amongst the research team to establish consensus.

### Trustworthiness

Trustworthiness was enhanced by keeping a reflexive journal throughout the research process, and included checking prior assumptions of the phenomena and the population, reviewing our position in the research, and any biases we hold [50]. An audit trail kept throughout the research process, documented each stage and decision, to provide a map of the evolution from raw data to findings and increase the study’s confirmability [51]. Member-checking and seeking consensus among the research team also contributed to trustworthiness in this study [46]. We adhered to the Standards for Reporting Qualitative Research (SRQR [52]) throughout this research.

## Findings

The first primary theme was “Safe Spaces, “ which is explored further in the “The Joys of Sharing” sub-theme. The second primary theme was “Not Safe to Share”, which included, with some overlap, two sub-themes, “The Perils of Sharing” and “Changing the Narrative”.

### Safe spaces

Participants unanimously reported being an “open book” and, when asked directly were happy to share their story in detail, provided they were in safe spaces, which consisted of people with similar views or experiences of birth. Sharing was dependent on ascertaining whether their audience was a safe space, “and then I would sort of drip-feed my story out while gauging their reactions” (Sarah). Safe spaces appeared more contingent on shared views and experiences than closeness, as many women felt they could not tell close friends and family members who held different views. One participant stated: “I’ve weirdly felt more comfortable sharing intimate pieces of my experience of a baby coming out of my vagina with relative strangers on social media, through relevant Facebook groups, who I know ‘get it’; than what I have with my best friends who’ve known me a lifetime” (Simone).

Dedicated online forums like birthing pages or doula business pages often provided a safe space to share. An important distinction was made between these dedicated forums and private Facebook pages which made participants feel vulnerable, “I just felt very like vulnerable sharing it with like friends, Facebook friends compared to people who have chosen to follow me in that business sense. Yeah, it felt safer to do that” (Helen). It was also safe to share with people who had no experience of birth as they held no frame of reference; however, one participant clarified that people who have not had a baby but view birth as negative are unsafe for sharing stories.

Many midwives regularly provided a safe space to share, as they were perceived as having similar views of birth:

“When my midwife came to see me afterwards because she really enjoyed it as well and I felt comfortable talking about it with her. And then, I have a couple of friends who have had positive experiences and I feel like I can talk about it with them, but not really anyone else” (Bronwyn).

These spaces were considered safe as they enabled participants to tell their stories free from criticism. Sharing their stories with like-minded people becomes an ingroup membership, where participants could celebrate their similarities and feel like they belonged. Participants reported sharing in a safe space was a joyful experience, explored in the sub-theme The Joys of Sharing.

#### The joys of sharing

Although it seemed relatively rare, participants enjoyed telling their birth stories when they found themselves in Safe Spaces. They are immensely proud of their stories and had delightful feelings come flooding back upon retelling, “Like I just felt incredible and I still, like talking about now, I still, like all of that comes back. … But I also, like all those feelings come flowing back. It’s like the oxytocin is still very high” (Helen). When participants felt they could not share or need to alter their stories to omit the positive aspects of their experience, they may have missed out on the feelings they report arise from sharing their authentic experience.

Participants felt validated in their experience when successfully sharing amongst like-minded people, “Yeah, it just, it’s relatable and it’s connecting and reaffirming of my own experience, that, ‘Oh, this isn’t just me like this is actually the power of it.’ Like it, yeah, mine wasn’t a one off. You know?” (Simone). The opportunity to align with the experience of others allowed participants to feel their experience is typical and to feel connected to others through shared storytelling. It allowed participants to process the details of their birth stories and justify decisions or reconcile aspects of their births they did not understand. Participants who shared in safe spaces felt they belonged to a positive birth movement, one they felt obligated to share with others to empower them to have the same.

Participants were unanimously passionate about sharing their stories to inspire others to have similar experiences, particularly after hearing only negative stories during their pregnancies and having to seek out positive birth stories actively:

“I love hearing them and I love telling my birth stories. I think it’s I think it’s a thing that women should bond over and that it takes us back to a simpler life where we were a village and everyone should share what happens to them, and we should build each other up and support each other and I do think that’s a big thing that’s missing from birthing, and parenting these days” (Kathleen).

Participants felt being informed, supported, autonomous in decisions, calm, and keeping a positive mindset all contributed to their positive experience, and felt obligated to share that with women to empower them to achieve a similar experience. Participants also felt their positive birth experiences improved the bond with their babies and positively impacted their ability and experience of parenting and were passionate about wanting that for other women. Empowering other women to strive towards these factors and achieve these positive outcomes contributed to motivations to share their stories:

“And the impact that could have on the world if all women emerged with that sense of power and confidence and euphoria and transformation and healing and all the words you know, what, what a difference it could make to the way we raise our children, and the way we continue to be women in the world” (Simone).

This quote illustrates the impact this participant believed positive births could have for women and why she believed sharing positive birth stories to empower women is important.

### Not safe to share

Participants wanted to speak about their births, but repeatedly felt unable to share their stories in a society they felt was dominated by a negative view of birth. Participants felt they were acutely aware of the dominant view of birth being negative, and reported they felt overwhelmed by the volume of “horror stories” they encountered during pregnancy. One participant said, “Because society suggests it shouldn’t be an enjoyable thing … There’s this push down from society. We shouldn’t enjoy birth, birth shouldn’t be a good thing, you know because, it’s a medical condition” (Kathleen). An awareness that their experience deviated from the norm made participants feel different, thus sharing their stories appeared to make them feel vulnerable and unsafe; “And I guess it sends a message then, sometimes when you have those experiences that it’s not okay and not safe to share such a positive story” (Simone).

Participants reported their motivation to participate became two-fold; to have the rare opportunity to share their story and contribute to research they hoped would change the dominant voice on birth. “Oh, I think probably just not seeing many avenues for actually being able to share positive birth stories. And because it was so positive, I feel, I feel so fulfilled by it that I, you know, when something’s wonderful you want to share, don’t you?” (Simone). This motivation was emphasised in the overwhelming response rate and echoed in the emails received from prospective participants. “I wanted to take part in this research because I think it’s important for ladies to know birth isn’t like what you see on the TV and it definitely [can] be an empowering, amazing experience.” (Milena).

Holding this positive view of birth appeared to thwart their ingroup membership with other parents, making them feel like a minority outgroup member. The vulnerability of sharing was strikingly evident in Mother’s Groups (a group of new mothers that is initially organised by community services in Western Australia), where that connection to other new mothers was vital to forming new relationships based on shared experiences. One participant explained:

“I think an initial mothers group going around in a circle and hearing everybody’s horrible experiences. I kind of felt that I couldn’t be too excited for myself because I didn’t want to hurt anybody else’s feelings, as silly as that sounds. But I guess that was, and because you don’t really know the group initially. So, you don’t want anyone to hate you straight away!” (Milena).

Mother’s groups also provide an opportunity for new parents to validate their birth experiences and feel normal in this pivotal life event, an opportunity participants felt they were denied, not solely based on the views of the other parents, but also the child health nurse facilitating the group, “I was just getting a little bit of blankness from other people and child health nurse, nurse was like facilitating it, she was like smiling and I mean it looked a little bit strained” (Helen). Participants explained this was a common experience with health providers, including some doctors and obstetricians. Having specialists in the field consider their experiences outside the norm provides authority to beliefs of being abnormal, potentially impacting participant’s well-being.

The sub-themes The Perils of Sharing and Changing the Narrative further explore situations when participants find themselves in situations deemed Not Safe to Share.

#### The perils of sharing

The perils of sharing were the adverse reactions of others that shaped participants’ experience of sharing their stories and contributed to feeling ostracised when speaking about birth. Participants were concerned about other’s feelings when sharing their birth stories, worried about being perceived as judging the experiences of others, “People seem defensive or when I was sharing my positive experience with her, she was very defensive and made out that I was trying to criticise her for having a caesarean when that’s not what I was doing at all” (Bronwyn). Birth stories are so unique and varied, and there seems to be a constant comparison and evaluation when sharing. People try to reconcile the differences between their stories and others to feel justified and validated in their own experiences. It appears that when stories are too different to reconcile nuances, women must attribute these differences by classifying people as outgroup members.

Participants were concerned sharing their birth stories would sound like bragging, “Oh, it makes you feel uncomfortable, like if I had been too, so that tall poppy syndrome stuff that’s like don’t be too big or too positive or too… because then you potentially look insensitive to someone else’s journey” (Simone). We argue that our Australian culture is a barrier to celebrating our achievements and feeling proud of ourselves. It is more accepted in Australian culture to focus on the negatives and challenges in stories than to revel in positivity and achievements, a sentiment captured by this participant, “Like you’re telling it because you know you had a lovely experience. It’s kind of like ‘Oh, I’ve got great hair today.’ Like yeah, yeah, good for you” (Stacey).

Participants found others to be disinterested in hearing their stories and found it stunted conversations. Reactions included awkward shock and eye rolls, being labelled as “hippie”, “radical”, or “crazy”, and many participants found people would directly challenge elements of their stories, attributing them to inaccurate recall or luck. These reactions highlight how participants were at risk of being stereotyped by their positive birth story. One participant declared, “I’m not radical. I’m not extreme or anything. I’m not going underground to give birth” (Alison). Another participant felt she was perceived as “Someone who lived outside society” (Sarah). The notion of being radical or living outside society is language indicative of someone with outgroup membership, but what does that mean for mothers when they feel they do not belong to motherland? Participants spoke about how they altered or withheld their stories to avoid this, discussed further in Changing the Narrative.

#### Changing the narrative

Participants were discerning about sharing their stories, to protect themselves and their experience, whilst being accepted by others; “It’s just like [a] positive pivotal point in your life. And like the last thing you want to do is for anyone to put a dent in it. You know you’re just, you’re so protective of that experience” (Sarah). Participants would choose not to share, or omit positive descriptors from their stories, merely describing the mechanics of the birth, offering a brief description without going into any detail. One participant described:

“It depends on the person and depends on what experiences they’ve had, because you sometimes have to temper what you say to people if they have not necessarily had a vaginal birth, or a positive birth… So, with people who are a lot more like pro-natural birth and stuff, I’m much happier to talk at-length, to use words like elated and amazing and remarkable and those kinds of things” (Stacey).

Changing the narrative allows participants to tell their stories in a way that aligns with the dominant view of birth, potentially allowing them access to ingroup membership and the opportunity to bond with other women over shared storytelling.

Participants spoke about justifying their stories by offering disclaimers or caveats. This gave participants the power of reconciling the differences in their stories, thus eliminating the need for others to attempt it. One participant felt compelled to include a negative aspect from parenting for balance:

“I said that like “Oh it was really beautiful” and then I immediately backed it up with “But unfortunately, even though like the birth went really smoothly, she’s been like, she’s been quite a colicky baby, so the, like it’s been really hard”, like I felt like I needed to put in that, like negative, to balance out things” (Helen).

By adapting their stories to fit the dominant view, participants compromise their own authentic identity, potentially impacting their well-being. Participants who could tell their story authentically in a safe space exhibited feelings of excitement and joy explored earlier in the theme Safe Spaces.

## Discussion

This study was the first to explore women’s experiences of sharing their positive birth stories to elucidate socially constructed motivations for or against sharing. Despite being open and excited to share their positive birth stories, the sample of 12 women often felt they were unable, as it challenged the dominant view of birth. The experienced and anticipated reactions from others made participants feel vulnerable and unsafe when sharing their stories, highlighting the differences of their experiences to the normative view of birth, making them feel like outsiders. Participants felt others invalidated their experiences by challenging details or attributing the experience to luck and would often protect themselves and their stories by changing the narrative. By withholding their stories, only sharing selected elements of their stories that fit the dominant view of birth or omitting positive descriptors and merely listing the mode of birth, participants mitigated the risk of outgroup membership. Participants enjoyed telling their birth stories in a safe space, which constituted people with similar experiences or views on birth, such as with many midwives, on dedicated Facebook forums, and with some close friends or family. Sharing their stories allowed women to access positive, euphoric feelings from their birth and feel reignited and elated. Sharing in safe spaces permitted participants to feel they belonged, an ingroup membership that created a space for validation of their own experiences. Participants felt motivated to share their stories to connect with and inspire other women to strive for positive birth experiences.

Reports that participants felt surrounded by an overwhelmingly negative portrayal of childbirth from others and in the media is supported by existing literature [2, 6], and contributed to feeling unable to share. Positive stories were considered a minority voice against the dominant view of birth, and participants felt othered by sharing their experience, which aligns with Social Identity Theory [42]. Considered through the lens of this theory, people who experience or subscribe to the dominant, negative view of birth forms a majority ingroup, and people who experience a positive birth are potentially rejected, becoming members of the motherland outgroup. Although the birth experience is more complicated than a mere dichotomy of positive and negative, it appears this perceived appraisal may categorise participants and potentially divide these women based on their group membership. Participants, therefore, felt apprehensive about sharing their stories, a phenomenon which may explain why participants themselves did not hear positive birth stories whilst pregnant and felt the need to seek them out, similar to the findings in Johnson et al. [3]

Participants’ reports of others challenging or invalidating their stories are consistent with Kay et al [12], who recounted the perspective of those hearing positive birth stories. In the theme “too perfect and wonderful: being economical with the truth” ([12] p5), women were quick to dismiss positive stories as they did not fit their preconceived understanding of childbirth, and therefore positive stories were not accepted as real-life experiences. Kay et al [12] also found their participants felt sharing positive birth stories would make other women feel like failures, by comparison, a common concern among participants in this study. These perceived and experienced reactions from others became a socially constructed understanding of sharing their birth stories, which influenced participants’ desire to withhold or alter them to fit the dominant narrative, reflected in the theme Changing the Narrative.

Participants’ motivation to withhold or part-tell their stories is compatible with Need-to-Belong Theory, which posits that people adapt their behaviours to seek approval from, and therefore connection to, others [41]. To achieve belongingness, people must have meaningful, non-negative interactions with others [41], a situation participants found not conducive to sharing their positive birth stories among people with opposing views. Baumeister [41] continued that self-esteem serves as a cognitive measure of our belongingness, so when we feel we belong, our self-esteem improves, and when we feel rejected, our self-esteem suffers. Future research may consider exploring a link between feeling unable to share positive birth stories and the effects on self-esteem. Perhaps this could account for our overwhelming response rate, and the motivation for participants to desire the opportunity to share their story in a safe space. Belongingness may hold additional importance, as Li et al [53] found that social support and connection to others in the perinatal period is associated with decreased rates of depression. Normalising the positive birth experience may provide greater opportunity for women to connect through sharing their birth stories.

The sub-theme The Joys of Sharing aligns with Johnson et al [3] findings who explored women’s motivations for sharing birth stories. They found women were motivated to share to build motherwisdom, a mutual exchange of information through sharing and listening to birth stories. They found the key motivators for building motherwisdom were normalising the experience for the self, empowering others, and validation of their own experience. When participants in the current study were in safe spaces, they too experienced validation of their experience and the desire to empower others, however, safe spaces were rare, and participants did not feel they had that opportunity very often. These findings suggest that participants are denied the normative experience of sharing birth stories most of the time, often deprived of the opportunity to receive validation of their own experiences, contribute to motherwisdom, and empower others.

Participants were very passionate about sharing their stories to empower future mothers to achieve the same positive experience. They attributed it to being informed, well supported, having autonomy over birthing decisions, trust in their body’s abilities, a positive mindset, and continuity of care, all of which is supported in the literature [8, 32, 35]. Interestingly, participants all had varied experiences, with stark differences in mode of birth, level of intervention, and care providers. Considering this and that only one participant described her birth as pain-free, emphasises that it is not a particular mode of birth that leads to a positive evaluation. Participants also felt compelled to share how their positive experience contributed to the bond and attachment they felt with their baby and their improved parenting experience, consistent with findings by Bell et al. [54] Future research may consider further exploration on factors that contribute to a positive appraisal of birth, including partner and caregiver support and autonomy over birthing decisions, and the long-term impact of these factors on the experience of parenting.

From a broader perspective, if women who experience positive births feel silenced, positive birth stories may only ever be considered in isolation, perpetuating a negative view of birth. This may overinflate the negative aspects of birthing, giving prospective mothers the idea that something will inevitably go wrong, driving them to make decisions based on a dependence on technology and medicalisation in pursuit of safety [12]. This moves away from focusing on autonomy and trust in their body’s natural ability to birth a child, two factors that contribute to a positive evaluation of birth [35]. A study on 350 pregnant women in Melbourne, Australia, found stories from family and friends were the third and fourth most common sources of information on childbirth, respectively, after midwives and an information pamphlet [38]. They found that 52.3% of women turned to family and 52.0% to friends as a source of their birthing information, higher than the internet (44.0%), antenatal education (42.6%) and obstetric advice (12.6%). This highlights the importance of birth stories as a source of information for prospective parents. Encouraging women to share their positive birth stories may contribute to normalising the positive birth experience, which may contribute to reducing fear of childbirth [6]. Normalising the positive birth experience may illuminate the possibility of that experience for women and empower them to strive towards achieving it for themselves, as participants in this study did.

### Limitations and future research

The unique exploration of the experiences of women telling their positive birth stories and its contribution to the growth of research on positive birth experiences is a strength of this research. A potential response bias may limit findings, as women who feel unable to share their positive birth stories may volunteer to participate to access an opportunity to tell their story. This may mean women with positive birth stories who have many avenues for sharing their stories did not feel the need to contribute. The sample was also relatively homogenous as women were tertiary-level educated Caucasians recruited within a narrow network. This demographic may have greater access to factors that promote a positive appraisal of birth, such as access to information, autonomy of decisions, and adequate support. All participants were from Western Australia and may not be reflective of the experiences of women from areas outside this region. We recommend future studies replicate this research with a diverse sample to strengthen transferability of findings and consider including a quantitative component to capture how many women perceive their births as positive and feel comfortable sharing their stories.

The inclusion of modes of birth that deviate from societal norms, such as home birthing, may influence reactions to participants’ birth stories, therefore skewing results and may be considered a limitation of this research. Future research may consider replication with the exclusion of non-hospital modes of birth; however, one could argue that the mode of birth contributes to the overall birth experience and is therefore relevant in sharing positive birth stories. The five participants in this study who delivered their baby at home attribute the calm, relaxed environment and autonomy of timeline and decisions to the positive evaluation of their experience.

We acknowledge ZN’s position as the researcher in this study as a potential limitation. ZN’s biases stem from her own positive birth and experiences sharing her birth story. Although efforts have been made throughout the research to mitigate this bias, such as reflexivity, audit trail, member-checking, and researcher consensus, it is impossible to entirely remove this subjectivity. ZN’s experience also enabled her to provide participants with a safe space for sharing their stories, potentially supporting women to feel comfortable enough to provide a rich description of their experiences, which may be deemed a strength of this research.

## Conclusion

This study explored the experience of women who shared their positive birth stories. Participants often felt that it was not safe to share their birth stories in a society dominated by a negative view of childbirth. When participants were in a safe space, with like-minded people, they experienced the same validation and joys of sharing that many women usually gain from sharing a typical birth story. Participants often had to withhold or part-tell their story to feel they belonged when sharing their stories. This may help to understand the lack of availability of positive birth stories in our society and why women feel they must actively seek them out. Understanding the motivations behind sharing or withholding their positive birth stories may contribute to finding avenues to normalise the positive birth experience, potentially contributing to changing society’s view of birth, which will hopefully improve birth experiences in the future.

### Relevance to clinical practice

One application of this research is developing an awareness of these experiences in perinatal care providers such as midwives, doctors, obstetricians, and child health nurses. Promoting a balanced view of childbirth at this authoritative level may encourage acceptance of positive birth stories, and validating stories during debriefing sessions may empower new mothers and facilitate acceptance. Antenatal education classes could also encourage portraying positive birth experiences and focus on educating women about the role of fear in childbirth and the importance of autonomy of decisions, information power, caregiver support, and faith in their body to deliver a baby.

At the systemic level, including positive birth experiences in the media may accurately portray childbirth as the varied experience it is, working towards a broader view of childbirth in society. Normalising the positive birth experience may potentially allow women with all experiences the opportunity to share their stories more openly by broadening safe spaces for sharing. It may also illuminate the possibility of a positive birth experience for women, and they may feel empowered to strive towards achieving it for themselves.

## Data Availability

The datasets generated and/or analysed during the current study are not publicly available due protecting participants privacy but are available from the corresponding author on reasonable request and with permission from participants.

## Abbreviations

ABC: Antecedents, behaviour, consequences
KP: Second author, Kelly Prandl
SRQR: Standards for reporting qualitative research
TAFE: Technical and further education
ZN: First author, Zaneta Neucom

## References

[1] Bilgin NC, Ak B, Ayhan F, Kocyigit F, Yorgun S, Topcuoglu MA (2020). Effect of childbirth education on the perceptions of childbirth and breastfeeding self-efficacy and the obstetric outcomes of nulliparous women. Health Care for Women International.

[2] Luce A, Cash M, Hundley V, Cheyne H, van Teijlingen E, Angell C (2016). "Is it realistic?" the portrayal of pregnancy and childbirth in the media. BMC Pregnancy Childbirth..

[3] Johnson NL, Scott SF, Brann M (2020). “Our birth experiences are what binds us”: Women’s motivations for storytelling about birth to build motherwisdom. Commun Stud.

[4] Dencker A, Nilsson C, Begley C, Jangsten E, Mollberg M, Patel H (2019). Causes and outcomes in studies of fear of childbirth: a systematic review. Women Birth.

[5] Roosevelt L, Low LK (2016). Exploring fear of childbirth in the United States through a qualitative assessment of the Wijma delivery expectancy questionnaire. J of Obstet, Gynecol, & Neonatal Nurs.

[6] Fisher C, Hauck Y, Fenwick J (2006). How social context impacts on women's fears of childbirth: a Western Australian example. Soc Sci Med.

[7] Jomeen J, Martin CR, Jones C, Marshall C, Ayers S, Burt K (2021). Tokophobia and fear of birth: a workshop consensus statement on current issues and recommendations for future research. J of Reprod and Infant Psychol..

[8] Greenfield M, Jomeen J, Glover L (2019). "It can't be like last time" - choices made in early pregnancy by women who have previously experienced a traumatic birth. Front Psychol.

[9] Kennedy HP, Nardini K, McLeod-Waldo R, Ennis L (2009). Top-selling childbirth advice books: a discourse analysis. Birth.

[10] Sanders RA, Crozier K (2018). How do informal information sources influence women's decision-making for birth? A meta-synthesis of qualitative studies. BMC Pregnancy Childbirth.

[11] Fleming SE, Vandermause R, Shaw M (2014). First-time mothers preparing for birthing in an electronic world: internet and mobile phone technology. J of Reprod and Infant Psychol..

[12] Kay L, Downe S, Thomson G, Finlayson K (2017). Engaging with birth stories in pregnancy: a hermeneutic phenomenological study of women's experiences across two generations. BMC Pregnancy and Childbirth..

[13] Allotey JC (2011). English midwives' responses to the medicalisation of childbirth (1671-1795). Midwifery.

[14] Newnham EC (2014). Birth control: power/knowledge in the politics of birth. Health Sociol Rev.

[15] Beckett K (2005). Choosing cesarean: feminism and the politics of childbirth in the United States. Fem Theory.

[16] Johanson R, Newburn M, Macfarlane A (2002). Has the medicalisation of childbirth gone too far?. BMJ..

[17] Hildingsson I, Nilsson C, Karlstrom A, Lundgren I (2011). A longitudinal survey of childbirth-related fear and associated factors. J of Obstetrics, Gynecol, & Neonatal Nurs.

[18] Stoll K, Edmonds JK, Hall WA (2015). Fear of childbirth and preferrence for cesarean delivery among young American women before childbirth: a survey study. Birth.

[19] Soderquist J, Wijma B, Thorbert G, Wijma K (2009). Risk factors in pregnancy for post-traumatic stress and depression after childbirth. BJOG.

[20] Beck CT, Watson S (2019). Mothers experiences interacting with infants after traumatic childbirth. MCN Am J Matern Child Nurs.

[21] Bell AF, Andersson E, Goding K, Vonderheid SC (2018). The birth experience and maternal caregiving attitudes and behavior: a systematic review. Sex Reprod Healthc.

[22] Gottvall K, Waldenstrom U (2002). Does a traumatic birth experience have an impact on future reproduction? BJOG: an international. J Obstet Gynaecol.

[23] Beck CT, Watson S (2010). Subsequent childbirth after a previous traumatic birth. Nurs Res.

[24] Ucar T, Golbasi Z (2019). Effect of an educational program based on cognitive behavioral techniques on fear of childbirth and the birth process. J Psychosom Obstet Gynaecol.

[25] Isbir GG, Inci F, Onal H, Yildiz PD (2016). The effects of antenatal education on fear of childbirth, maternal self-efficacy and post-traumatic stress disorder (PTSD) symptoms following childbirth: an experimental study. Appl Nurs Res.

[26] Karabulut Ö, Coşkuner Potur D, Doğan Merih Y, Mutlu SC, Demirci N (2016). Does antenatal education reduce fear of childbirth?. Int Nurs Rev.

[27] Fenwick J, Toohill J, Gamble J, Creedy DK, Buist A, Turkstra E (2015). Effects of a midwife psycho-education intervention to reduce childbirth fear on women's birth outcomes and postpartum psychological wellbeing. BMC Pregnancy Childbirth..

[28] Streibert LA, Reinhard J, Yuan J, Schiermeier S, Louwen F (2015). Clinical study: change in outlook towards birth after a midwife led antenatal education programme versus hypnoreflexogenous self-hypnosis training for childbirth. Geburtshilfe Frauenheilkd.

[29] Hong K, Hwang H, Han H, Chae J, Choi J, Jeong Y (2021). Perspectives on antenatal education associated with pregnancy outcomes: systematic review and meta-analysis. Women Birth..

[30] Brixval CS, Axelsen SF, Lauemoller SG, Andersen SK, Due P, Koushede V (2015). The effect of antenatal education in small classes on obstetric and psycho-social outcomes: a systematic review. Syst Rev.

[31] Catling CJ, Medley N, Foureur M, Ryan C, Leap N, Teate A, et al. Group versus conventional antenatal care for women. Cochrane Database Syst Rev. 2015:CD007622. 10.1002/14651858.CD007622.pub3. 10.1002/14651858.CD007622.pub3 PMC6465187 25922865

[32] Thomson GM, Downe S. Changing the future to change the past: Women’s experiences of a positive birth following a traumatic birth experience. J of Reprod and Infant Psychol. 2010;28(1):102–112. p. 102. doi:10.1080/02646830903295000.

[33] Aune I, Marit Torvik H, Selboe ST, Skogas AK, Persen J, Dahlberg U (2015). Promoting a normal birth and a positive birth experience - Norwegian women's perspectives. Midwifery..

[34] Quine L, Rutter DR, Gowen S (1993). Women's satisfaction with the quality of the birth experience: a prospective study of social and psychological predictors. J of Reprod and Infant Psychol.

[35] Karlstrom A, Nystedt A, Hildingsson I (2015). The meaning of a very positive birth experience: focus groups discussions with women. BMC Pregnancy and Childbirth.

[36] Dahlen HG, Barclay LM, Homer CS (2010). Processing the first birth: journeying into 'motherland'. J Clin Nurs.

[37] de Quattro L (2019). Co-producing childbirth knowledge: a qualitative study of birth stories in antenatal sessions. BMC Pregnancy Childbirth..

[38] Grimes HA, Forster DA, Newton MS (2014). Sources of information used by women during pregnancy to meet their information needs. Midwifery..

[39] Callister LC (2004). Making meaning: Women's birth narratives. J Obstet Gynecol Neonatal Nurs.

[40] Pennebaker JW, Seagal JD. Forming a story: the health benefits of narrative. J Clin Psychol. 1999;55(10):1243–54. https://pubmed.ncbi.nlm.nih.gov/11045774/. 10.1002/(SICI)1097-4679(199910)55:10<1243::AID-JCLP6>3.0.CO;2-N 11045774

[41] Baumeister RF, Lange PAV, Kruglanski AW, Higgins ET (2011). Need-to-belong theory. Handbook of theories of social psychology.

[42] Ellemers N, Haslam SA, Van Lange PAM, Kruglanski AW, Higgins ET (2012). Social identity theory. Handbook of theories of social psychology.

[43] Kukla A, Newton-Smith WH (2000). Social constructivism and the philosophy of science.

[44] Malterud K, Siersma VD, Guassora AD (2016). Sample size in qualitative interview studies: guided by information power. Qual Health Res.

[45] Rodriguez-Almagro J, Hernandez-Martinez A, Rodriguez-Almagro D, Quiros-Garcia JM, Martinez-Galiano JM, Gomez-Salgado J (2019). Women's perceptions of living a traumatic childbirth experience and factors related to a birth experience. Int J Environ Res Public Health.

[46] Ryan F, Coughlan M, Cronin P (2007). Step by step guide to critiquing research. Part 2: qualitative research. Br J Nurs.

[47] Hoffart N (1991). A member check procedure to enhance rigor in naturalistic research. West J Nurs Res.

[48] Clarke V, Braun V, Hayfield N, Smith JA (2015). Thematic analysis. Qualitative psychology.

[49] Braun V, Clarke V (2006). Using thematic analysis in psychology. Qual Res Psychol.

[50] Guillemin M, Gillam L (2016). Ethics, reflexivity, and “ethically important moments” in research. Qual Inq.

[51] Carcary M (2009). The research audit trail: enhancing trustworthiness in qualitative enquiry. Electron J on BusResearch Methods.

[52] O'Brien BC, Harris IB, Beckman TJ, Reed DA, Cook DA (2014). Standards for reporting qualitative research: a synthesis of recommendations. Acad Med.

[53] Li Y, Long Z, Cao D, Cao F (2017). Social support and depression across the perinatal period: a longitudinal study. J Clin Nurs.

[54] Bell AF, Rubin LH, Davis JM, Golding J, Adejumo OA, Carter CS (2019). The birth experience and subsequent maternal caregiving attitudes and behavior: a birth cohort study. Arch of Women's Mental Health.

